# Supplementary material for: The longitudinal association of stressful life events with depression remission among SHARP trial participants with depression and hypertension or diabetes in Malawi
Source: PLoS One. 2024 Feb 26;19(2):e0298546. doi: 10.1371/journal.pone.0298546 (PMC10896523; doi:10.1371/journal.pone.0298546)
Supplement: Attachment — Submitted filename: response_to_reviewers.docx [file pone.0298546.s004.docx]

Dear PLOS ONE Editors and Reviewers,

Thank you so much for your time and responses. We greatly appreciate your thoughtful questions and comments and we hope that these changes address all of your concerns. Please find our responses in line below and the corresponding revisions in the revised manuscript.

2. Thank you for stating the following financial disclosure:

"This study received funding from the National Institute of Mental Health (MPIs: Brian W. Pence, PhD, Mina C. Hosseinipour, MD MPH; Jones Masiye; U19MH113202-01). KRL received support from supplement U19MH113202-04S1. JMD received support from supplement U19MH113202-01S2 and institutional training grant T32AI070114. No funding agency or funder had any part in study conceptualization, data collection, data analysis, manuscript preparation, or publication decision-making."

Please state what role the funders took in the study.  If the funders had no role, please state: "The funders had no role in study design, data collection and analysis, decision to publish, or preparation of the manuscript."

*"This study received funding from the National Institute of Mental Health (MPIs: Brian W. Pence, PhD, Mina C. Hosseinipour, MD MPH; Jones Masiye; U19MH113202-01). KRL received support from supplement U19MH113202-04S1. JMD received support from supplement U19MH113202-01S2 and institutional training grant T32AI070114. The funders had no role in study design, data collection and analysis, decision to publish, or preparation of the manuscript."*

If this statement is not correct you must amend it as needed.

Please include this amended Role of Funder statement in your cover letter; we will change the online submission form on your behalf.

Additional Editor Comments:

Thank you for the opportunity to serve as the Editor for the manuscript titled "The longitudinal association of stressful life events with depression Remission among patients with depression and Hypertension or diabetes in Malawi". The author's work for the manuscript is commendable. However, the manuscript looks statistically sound but methodologically weak. I have a lots of major concerns that need to be addressed to make this manuscript an important one for the readers of this journal and consider acceptance

General considerations:

Kindly spell check the entire manuscript for grammatical errors and use of punctuation

*We have further proofread the manuscript for technical, grammatical, and punctuation errors. Thank you.*

Title:
Consider adding the study design to the title

*The title, “The longitudinal association of stressful life events with depression remission among SHARP trial participants with depression and hypertension or diabetes in Malawi”, describes that this is a longitudinal analysis. We have added “among a cohort” to clearly show that this is analysis is situated in the SHARP RCT.*

Abstract:
Please stick to the journal guidelines for abstract preparation

Introduction:
1. Introduction's first paragraph is highly confusing. It is, however, not clear what estimates the authors are providing in the first paragraph. On depression or NCDs. Be specific in providing the estimates - using a funnel approach - burden globally, in LMICs and then in Malawi - for NCD, SLEs, depression, its association and the possible effect of SLE on remission

*Thank you for this comment. We have edited the introduction accordingly. We agree that this approach makes it clearer to the reader that this is a study of the longitudinal association between depression and SLEs among patients that have depression (who also happen to have hypertension and/or diabetes, though NCDs are not the focus of the study themselves.)*

2. Too many references quoted in the first two paragraphs, there are almost 20 references quoted in the first 2 paragraphs. This opens up a question is so much literature is available why do a primary study again?

*Thank you for this comment. After rearranging and editing the introduction, we hope that this addresses this comment. We conducted a thorough review of the literature (global, nationally, and sub-nationally)*

3. Please mention the rationale and novelty of the study and what the study results are expected to add

*We have extended this section of the introduction, thank you for this comment.*

Methods and Results:
1. Please elaborate more on the study setting, explaining the mental health and NCD care context that exists in Malawi. Service provision and the care pathway that exists for the treatment of a patient with NCD or depression

*We have elaborated on the context of mental health care service provision, including where the SHARP trial is situated in integrated mental health and NCD service provision in the country. We have added this to expand the introduction, and further expand the introduction according to the above comments.*

2. Did the authors obtain the necessary permission for the use of data from the SHARP study

*Yes, the authors are SHARP study team members.*

3. The parent study is also authored by the same authors? Please declare the COI - if any for the use of data from the previous study

*There are a multitude of published manuscripts from the SHARP trial, with some of the same coauthors as this manuscript. There are not conflicts of interest, as declared by all authors during manuscript submission. Funding sources and agencies had no role in study conceptualization, data collection, data analyses, manuscript preparation or reporting, or any other study processes.*

4. How did the authors obtain a cohort from an RCT? The RCT has a controlled environment? can a cohort mimicking natural history be taken from an RCT?

*All data are drawn from the prospective RCT. After accounting for study site and arm in regression analyses, this allows us to look at a cohort of RCT patients prospectively and longitudinally. We have added more discussion regarding this. Thank you for bringing up this point. We hope that our clarifications address this concern.*

5. Please explain more on the acquisition of data

*As also described above, we have elaborated on data ascertainment in the Methods section. The study instruments are described in the Measures section of the Methods section. Given that we are the research team for the SHARP study, we collect, manage, and report the data ourselves.*

6. What is exactly the study design? is it a cohort? or a plain observational analysis?
7. This line "Those participants (n=203) who had a PHQ-9 score ≥5 at screening and consent but a PHQ-9 score <5 in the baseline interview were excluded from this analysis" is highly misleading and contradicts the inclusion criteria. Please be specific and clear

*Thank you for this comment. We have clarified this in this manuscript section.*

*“Participants who were consented and enrolled in the parent study (n=203) who had a PHQ-9 score ≥5 at screening, but a PHQ-9 score <5 in the baseline interview, were excluded from this analysis.*^1^ *While at screening, these participants were eligible for participation, we do not know why their PHQ-9 score, which should be relatively stable over a short period of time, was lower during the baseline interview.”*

8. Please elaborate more on the SLE questionnaire, if it's a validated instrument.? and how did the authors arrive at the specified categorisation?

*Yes, the LES is a validated instrument for assessment of 57 SLEs in adults.*^2–4^ *The instrument was adapted to address categories of SLEs specific to this study population: experiencing death or illness of a friend or family member; hospitalization; a major new health problem; experiencing assault, robbery, and/or a motor vehicle accident; financial stress (measured by number of days going to bed hungry in the last month); employment changes and challenges; and relationship changes (e.g., marriage, divorce, and estrangement).*^2,3,5,6^ *The original LES survey includes SLEs that are not relevant in this context and that were excluded from our survey: foreclosure on mortgage or loan and student related SLES (e.g., failing an important exam, failing a course, dropping a course).* ^2–4^

*Thank you for this comment. We have provided additional information and rationale for decision making.*

9. Did the authors attempt to adjust for the possible mediators using the mediation analysis?

*We did not conduct a mediation analysis. Anxiety and PTSD may be mediators between SLEs and depression remission, and including adjusting for these variables in our current would result in biased estimates. When included in the primary analysis model (e.g., at the 3mo time point), inclusion of anxiety as a confounder changes the effect estimate substantially (~10% increase in the adjusted CIR) while inclusion of ptsd does not change the effect estimate substantially (<5% increase in the adjusted CIR). A formal mediation analysis is beyond the scope of this paper, but there is evidence for anxiety and ptsd being associated with stressful life events and depression (or lack of depression remission in our case, as they often occur comorbidly).*^7–13^

10. SLEs 3 moths prior to the interviews were linked with remission of depression recorded during the time of the interview? Was the sufficient theoretically possible time for the development of depression following SLEs considered before associating SLE and depression remission?

*Thank you for this comment. At each interview, participants were asked about SLEs experience in the three months BEFORE/LEADING UP TO that interview. So, at 3 months, participants were asked about SLES in the three months prior to that interview (the three months after the baseline interview and before the 3 month follow up interview). We have ensured clarification of this in the manuscript and hope that this addresses these questions.*

11. In case the patients reported SLE a day prior to interview and is found to have depression right now and no depression during follow-ups.. how was such a scenario handled in the manuscript?

*This is an interesting question, thank you for raising this point. It is possible that the participant experienced a SLE 1 day before the interview, but screens negative for depression. This participant would be considered as not having depression at that interview. The impact of SLEs can vary across and within individuals over time, so it is possible they experienced an SLE but are not experiencing depression symptoms at that time point.*^14–18^

12. Why SLE score of 2 taken as separate category?

*The SLE measure is a continuous measure. As such, we report the mean (SD), median, minimum, and maximum values for participants at baseline. We have also reported SLEs as a categorical variable, based on quartiles of the continuous SLE distribution (≥0, <2; ≥2, <3; ≥3, <5; ≥5, <15)In our results (Table 3), we report change in CIR per SLE, and per 3 SLEs (the median number of SLEs experienced in the study cohort).*

13. There is no mention o Table 5 in the manuscript

*Thank you for this comment, this should have said Table 4 and is now corrected.*

14. Was the effect of COVID-19 on the incidence of depression and the SLE adjusted in the analysis?

*We cannot measure incident depression in this analysis, as all participants have depression at study baseline (therefore, there can be no new, incident cases diagnosed). Further, we could not have anticipated having to do this additional analysis during the SHARP study’s design, as we did not anticipate having to measure variables related to a pandemic. As such, we assess if the CIR of depression remission changes before and during the pandemic. We report mean PHQ-9 scores before and during the pandemic, number and proportion of each type of SLE before and during the pandemic, and CIRs before and during the pandemic. We hope this answers your question, thank you for this point.*

Discussion:
The discussion looks very weak and poorly written. It is not enough when we plainly mention similar studies, rather efforts need to be made to compare and contrast the results and findings across the study settings. Please rewrite the discussion part discussing more on: i) comparison with previous studies ii) biological or theoretical reasons for the estimates obtained iii) Clinical implications iv) Programmatic or policy implications
Many references are not in Vancouver format

*Thank you for these comments. We have further expanded comparison of study results to other studies and hope that this sufficiently addresses your concerns.*

[Note: HTML markup is below. Please do not edit.]

Reviewers' comments:

Reviewer's Responses to Questions

**Comments to the Author**

1. Is the manuscript technically sound, and do the data support the conclusions?

The manuscript must describe a technically sound piece of scientific research with data that supports the conclusions. Experiments must have been conducted rigorously, with appropriate controls, replication, and sample sizes. The conclusions must be drawn appropriately based on the data presented.

Reviewer #1: Yes

Reviewer #2: Yes

2. Has the statistical analysis been performed appropriately and rigorously?

Reviewer #1: I Don't Know

Reviewer #2: Yes

3. Have the authors made all data underlying the findings in their manuscript fully available?

The [PLOS Data policy](http://www.plosone.org/static/policies.action#sharing) requires authors to make all data underlying the findings described in their manuscript fully available without restriction, with rare exception (please refer to the Data Availability Statement in the manuscript PDF file). The data should be provided as part of the manuscript or its supporting information, or deposited to a public repository. For example, in addition to summary statistics, the data points behind means, medians and variance measures should be available. If there are restrictions on publicly sharing data—e.g. participant privacy or use of data from a third party—those must be specified.

Reviewer #1: Yes

Reviewer #2: Yes

4. Is the manuscript presented in an intelligible fashion and written in standard English?

PLOS ONE does not copyedit accepted manuscripts, so the language in submitted articles must be clear, correct, and unambiguous. Any typographical or grammatical errors should be corrected at revision, so please note any specific errors here.

Reviewer #1: Yes

Reviewer #2: Yes

5. Review Comments to the Author

Please use the space provided to explain your answers to the questions above. You may also include additional comments for the author, including concerns about dual publication, research ethics, or publication ethics. (Please upload your review as an attachment if it exceeds 20,000 characters)

Reviewer #1: The authors have selected an interesting topics. The conclusion is supported by the results. Addressing stressful life events during integrated non communicable disease and depression care interventions can improve success of depression treatment among adult patients.

*Thank you for your comments and time in reading this manuscript. We hope that, collective with all comments and questions, we have improved the manuscript to address all concerns and strengthen the manuscript as a whole.*

Minor comnents:- you have used old references eg reference 25,50,51,52

*Thank you for this point. Reference 25 refers to the original PHQ-9 citation (the instrument itself). We also provide validation studies for the PHQ-9 tool for use in this context. References 50-52 is are key early studies in SLE and depression research, and cover a large scope of foundational research on this topic. We hope that, with more recent SLE and depression studies cited and added, this is sufficient to provide a review of what is currently known (and unknown) in the depression and SLE field of research.^19–24^*

Reviewer #2: Hello. Thank you for the opportunity to review this interesting article. I think it was an valuable endeavor and done quite well in the face of the pandemic and other barriers.
I understand the conclusion suggests that recent SLEs affect time to remission for depression. Although, that does sounds reasonable , there are a few questions I had in this regard-
1) What was the treatment modality employed to treat the depression? Was there any variation between the different groups in terms of what depression modality was used which could confound the results. You can mention what the treatment was in the methods.
2) Was there a difference in the hypertension treatment/ actually blood pressure control of the two studied groups. That could also present a variable that might confound.
3) The final conclusion you came to was the screening for SLEs might help in mitigating impact on depression severity and improving treatment- How? Again, we don't know what was used to treat the patients so we can't comment on how knowing SLEs might affect the treatment or the treatment choices. They might affect expectations of remission for the clinicians. You could elaborate on this in your conclusion or in your strengths/ limitations. I think depression screening would definitely be helpful but have to elaborate on the helpfulness of SLE screening.

*Thank you very much for your comments and for your time in reviewing our manuscript, and helping strengthen our reporting of this study’s results. Please find our responses in line below. We hope that they sufficiently address your questions and concerns.*

1) What was the treatment modality employed to treat the depression? Was there any variation between the different groups in terms of what depression modality was used which could confound the results. You can mention what the treatment was in the methods.

*Thank you for this comment. We have added discussion of this to the methods section accordingly. Clinics were randomized to one of two depression screening and treatment strategies, either a basic or enhanced implementation package. The basic implementation package involves a trained provider supporting SHARP implementation. The enhanced implementation package involves a trained provider supporting the intervention and a quality assurance program.*

*Participants receive standard of care in both packages. The SHARP intervention recommended the following, though treatment decisions are the decision of the provider and each patient: PHQ-9 score 0-4 does not have recommended treatment, if the patient has a score of 5-9 it is recommended that they receive the Friendship Bench (FB) intervention, and if the patient has a PHQ-9 score of 10 or higher it is recommended that they consider prescription depression treatment options.*^1,25^ *The FB intervention is a validated peer-counseling intervention, which has been validated for use in Malawi and Zimbabwe and is currently being assessed for use in Hanoi, Vietnam.*^1,26,27^

2) Was there a difference in the hypertension treatment/ actually blood pressure control of the two studied groups. That could also present a variable that might confound.

*This is a very interesting point. Hypertension treatment was based on provider expertise and patient-provider decision making. We believe that, though hypertension and cardiovascular disease may be linked to depression* ^19–23^*, hypertension is a mediator on the pathway from SLEs to depression or depression remission. SLEs and chronic stress can lead to cardiovascular diseases and are also related to hypertension, which may impact depression and depression remission.*^24^

3) The final conclusion you came to was the screening for SLEs might help in mitigating impact on depression severity and improving treatment- How? Again, we don't know what was used to treat the patients so we can't comment on how knowing SLEs might affect the treatment or the treatment choices. They might affect expectations of remission for the clinicians. You could elaborate on this in your conclusion or in your strengths/ limitations. I think depression screening would definitely be helpful but have to elaborate on the helpfulness of SLE screening.

*These are excellent comments and points, thank you. The rational for this statement is that depression is frequently undiagnosed in the study context, and identifying patients at particularly high risk for depressive disorders may increase detection and treatment of depression cases that otherwise would have been missed. Depression diagnoses are often missed in NCD clinic settings in Malawi, and knowledge of recent SLEs may help identify patients at risk of depression, particularly TRD.*^28^ *We hope that our expansion of this discussion is suitable to addressing your comment, thank you again for raising these points.*

6. PLOS authors have the option to publish the peer review history of their article ([what does this mean?](https://journals.plos.org/plosone/s/editorial-and-peer-review-process#loc-peer-review-history)). If published, this will include your full peer review and any attached files.


If you choose “no”, your identity will remain anonymous but your review may still be made public.


**Do you want your identity to be public for this peer review?** For information about this choice, including consent withdrawal, please see our [Privacy Policy](https://www.plos.org/privacy-policy).

Reviewer #1: **Yes:** Agmas Wassie Abate

Reviewer #2: No

[NOTE: If reviewer comments were submitted as an attachment file, they will be attached to this email and accessible via the submission site. Please log into your account, locate the manuscript record, and check for the action link "View Attachments". If this link does not appear, there are no attachment files.]

While revising your submission, please upload your figure files to the Preflight Analysis and Conversion Engine (PACE) digital diagnostic tool, <https://pacev2.apexcovantage.com/>. PACE helps ensure that figures meet PLOS requirements. To use PACE, you must first register as a user. Registration is free. Then, login and navigate to the UPLOAD tab, where you will find detailed instructions on how to use the tool. If you encounter any issues or have any questions when using PACE, please email PLOS at [figures@plos.org](mailto:figures@plos.org). Please note that Supporting Information files do not need this step.

*References*

*1.	Gaynes, B. N. et al. The Sub-Saharan Africa Regional Partnership (SHARP) for Mental Health Capacity-Building Scale-Up Trial: Study Design and Protocol. Psychiatr Serv* ***72****, 812–821 (2021).*

*2.	Sarason, I. G., Johnson, J. H. & Siegel, J. M. Assessing the impact of life changes: Development of the Life Experiences Survey. Journal of Consulting and Clinical Psychology* ***46****, 932–946 (1978).*

*3.	Sarason, I. G. & Johnson, J. H. The Life Experiences Survey: Preliminary Findings. https://apps.dtic.mil/sti/citations/ADA027527 (1976).*

*4.	Reif, S. et al. Highly Stressed: Stressful and Traumatic Experiences among individuals with HIV/AIDS in the Deep South. AIDS Care* ***23****, 152–162 (2011).*

*5.	Stack, R. J. & Meredith, A. The Impact of Financial Hardship on Single Parents: An Exploration of the Journey From Social Distress to Seeking Help. Journal of Family and Economic Issues* ***39****, 233–242 (2018).*

*6.	Guan, N., Guariglia, A., Moore, P., Xu, F. & Al-Janabi, H. Financial stress and depression in adults: A systematic review. PLoS One* ***17****, e0264041 (2022).*

*7.	Kendler, K. S., Hettema, J. M., Butera, F., Gardner, C. O. & Prescott, C. A. Life Event Dimensions of Loss, Humiliation, Entrapment, and Danger in the Prediction of Onsets of Major Depression and Generalized Anxiety. Arch Gen Psychiatry* ***60****, 789–796 (2003).*

*8.	Michl, L. C., McLaughlin, K. A., Shepherd, K. & Nolen-Hoeksema, S. Rumination as a mechanism linking stressful life events to symptoms of depression and anxiety: Longitudinal evidence in early adolescents and adults. Journal of Abnormal Psychology* ***122****, 339–352 (2013).*

*9.	Kim, J. I., Park, H. & Kim, J.-H. The mediation effect of PTSD, perceived job stress and resilience on the relationship between trauma exposure and the development of depression and alcohol use problems in Korean firefighters: A cross-sectional study. Journal of Affective Disorders* ***229****, 450–455 (2018).*

*10.	Lowe, S. R. et al. Pathways from assaultive violence to post-traumatic stress, depression, and generalized anxiety symptoms through stressful life events: longitudinal mediation models. Psychological Medicine; Cambridge* ***47****, 2556–2566 (2017).*

*11.	Buccheri, T., Musaad, S., Bost, K. K. & Fiese, B. H. Development and assessment of stressful life events subscales – A preliminary analysis. Journal of Affective Disorders* ***226****, 178–187 (2018).*

*12.	Weinberg, A., Kujawa, A. & Riesel, A. Understanding Trajectories to Anxiety and Depression: Neural Responses to Errors and Rewards as Indices of Susceptibility to Stressful Life Events. Curr Dir Psychol Sci* ***31****, 115–123 (2022).*

*13.	Flory, J. D. & Yehuda, R. Comorbidity between post-traumatic stress disorder and major depressive disorder: alternative explanations and treatment considerations. Dialogues Clin Neurosci* ***17****, 141–150 (2015).*

*14.	Hammen, C. Risk factors for depression: An autobiographical review. Annu. Rev. Clin. Psychol.* ***14****, 1–28 (2018).*

*15.	Kendler, K. S., Thornton, L. M. & Gardner, C. O. Stressful Life Events and Previous Episodes in the Etiology of Major Depression in Women: An Evaluation of the “Kindling” Hypothesis. AJP* ***157****, 1243–1251 (2000).*

*16.	Monroe, S. & Harkness, K. L. Life Stress, the ‘Kindling’ Hypothesis, and the Recurrence of Depression: Considerations From a Life Stress Perspective. Psychological Review* ***112****, 417–445 (2005).*

*17.	Post, R. M. Transduction of psychosocial stress into the neurobiology of recurrent affective disorder. AJP* ***149****, 999–1010 (1992).*

*18.	Stroud, C. B., Davila, J., Hammen, C. & Vrshek-Schallhorn, S. Severe and nonsevere events in first onsets versus recurrences of depression: Evidence for stress sensitization. Journal of Abnormal Psychology* ***120****, 142–154 (2011).*

*19.	Cohen, S., Murphy, M. L. M. & Prather, A. A. Ten Surprising Facts About Stressful Life Events and Disease Risk. Annu Rev Psychol* ***70****, 577–597 (2019).*

*20.	Cohen, S., Gianaros, P. J. & Manuck, S. B. A Stage Model of Stress and Disease. Perspect Psychol Sci* ***11****, 456–463 (2016).*

*21.	Dimsdale, J. E. Psychological Stress and Cardiovascular Disease. Journal of the American College of Cardiology* ***51****, 1237–1246 (2008).*

*22.	Steptoe, A. & Kivimäki, M. Stress and cardiovascular disease. Nature Reviews. Cardiology* ***9****, 360–370 (2012).*

*23.	Netterstrøm, B. et al. The Relation between Work-related Psychosocial Factors and the Development of Depression. Epidemiologic Reviews* ***30****, 118–132 (2008).*

*24.	Berntson, J., Patel, J. S. & Stewart, J. C. Number of recent stressful life events and incident cardiovascular disease: Moderation by lifetime depressive disorder. Journal of Psychosomatic Research* ***99****, 149–154 (2017).*

*25.	Akiba, C. F. et al. The Sub-Saharan Africa Regional Partnership (SHARP) for Mental Health Capacity Building: a program protocol for building implementation science and mental health research and policymaking capacity in Malawi and Tanzania. International Journal of Mental Health Systems vol. 13 1–13 https://doaj-org.libproxy.lib.unc.edu (2019).*

*26.	Chibanda, D. et al. Effect of a Primary Care–Based Psychological Intervention on Symptoms of Common Mental Disorders in Zimbabwe: A Randomized Clinical Trial. JAMA* ***316****, 2618–2626 (2016).*

*27.	Stockton, M. A. et al. The impact of an integrated depression and HIV treatment program on mental health and HIV care outcomes among people newly initiating antiretroviral therapy in Malawi. PLoS One* ***15****, e0231872 (2020).*

*28.	Udedi, M., Pence, B. W., Stewart, R. C. & Muula, A. S. Detection and prevalence of depression among adult type 2 diabetes mellitus patients attending non-communicable diseases clinics in Lilongwe, Malawi. International Journal of Mental Health Systems (2020) doi:http://dx.doi.org/10.21203/rs.3.rs-39726/v2.*
